# Online group therapies for anxiety, obsessive-compulsive, and trauma-related disorders: a systematic review

**DOI:** 10.3389/fnhum.2023.1286865

**Published:** 2024-01-11

**Authors:** Luana D. Laurito, Samara dos Santos-Ribeiro, Maria E. Moreira-de-Oliveira, Carla P. Loureiro, Verônica Hühne, Bianca Torres, Livi Ferreira Testoni de Faro, Gabriela B. de Menezes, Leonardo F. Fontenelle

**Affiliations:** ^1^Obsessive, Compulsive, and Anxiety Spectrum Research Program, Institute of Psychiatry, Federal University of Rio de Janeiro, Rio de Janeiro, Brazil; ^2^D’Or Institute for Research and Education, Rio de Janeiro, Brazil; ^3^Department of Psychiatry, Monash University, Clayton, VIC, Australia

**Keywords:** anxiety, trauma, obsessive-compulsive, online group therapies, internet-based therapy, video teleconference

## Abstract

**Background:**

This systematic review examined the existing literature to determine the evidence supporting the efficacy of online group treatments for anxiety-, obsessive-compulsive- and trauma-related disorders (AOTDs).

**Methods:**

A systematic review using the PUBMED, PsycInfo, Web of Science, and ClinicalTrials databases with no language, date, or study design filters was performed. The inclusion criteria comprised studies that examined individuals who had received a formal diagnosis of AOTDs, were aged 18 years or older, and had baseline and endpoint assessments of symptom severity using formal tools.

**Results:**

Five studies on social anxiety disorder (SAD), four on post-traumatic stress disorder (PTSD) and one on tic disorders (TDs) were found. The studies were open-label (*n* = 2) and randomized controlled trials (RCTs) (*n* = 8), with five of the RCTs being non-inferiority trials. Most studies were conducted in the US and investigated psychological CBT based interventions via internet-based therapies (IBT: *n* = 4), video teleconferencing (VTC: *n* = 5) or a combination of both (*n* = 1). In SAD, IBT studies associated with a clinician assisted web-based forum (here termed “forum-enhanced” studies) were superior to waiting lists and not inferior to similar versions that were also “forum enhanced” but self-guided, “telephone enhanced” by a contact with a non-specialist, and “email enhanced” by a contact with a clinician individually. Studies involving VTC have shown comparable effectiveness to in-person interventions across some online group CBT based treatments for PTSD. Two open trials also demonstrated symptoms reductions of social anxiety and tics through VTC.

**Conclusion:**

There is evidence supporting the effectiveness of online group treatments for SAD and PTSD. Further studies from different research groups may be needed to replicate the use of these and other forms of online treatments in individuals with SAD, PTSD, and other clinical populations, such as OCD, panic disorder, agoraphobia and specific phobias.

**Systematic review registration:**

https://www.crd.york.ac.uk/prospero/, identifier CRD42023408491.

## 1 Introduction

Anxiety disorders (ADs), obsessive-compulsive and related disorders (OCRDs) and trauma related disorders (TRDs) are highly prevalent mental health conditions that impose significant distress on individuals and society as a whole (Kessler et al., 2009; Ruscio et al., 2010; Stein et al., 2017). A systematic review indicates that the global prevalence of anxiety disorders, as defined by the fourth edition of Diagnostic and Statistical Manual of Mental Disorders (DSM-IV), is estimated to be 7.3%, with a range of 4.8 to 10.9% around the globe (Baxter et al., 2013; Stein et al., 2017). However, this prevalence does not encompass certain disorders mentioned earlier under the DSM-5 criteria. Collectively referred here as AOTDs, these disorders are characterized by different levels of anxiety or fear; intrusive thoughts, images, or memories; and avoidant or compulsive behaviors that cause significant distress and impact daily functioning (American Psychiatric Association, 2013).

Despite the availability of evidence-based treatments, such as antidepressant medications (Bandelow et al., 2012; Koran and Simpson, 2013; Baldwin et al., 2014; Brakoulias et al., 2016) and cognitive-behavioral therapy (CBT) (Abramowitz et al., 2018; Hezel and Simpson, 2019), a significant number of AOTDs patients remain untreated or continue to experience symptoms and impaired quality of life (Brakoulias et al., 2019). Factors such as long distances scarcity of specialized therapists, lengthy waiting lists, and financial constraints act as barriers to individuals seeking traditional therapies (Andrade et al., 2014). Because AOTDs patients may remain housebound due to severe avoidant behaviors, telemedicine may provide an initial therapeutic contact or a reasonable alternative to in-person treatment for individuals that would otherwise remain untreated.

Although telemedicine is not new, and teletherapies has been around for at least 20 years (Weinberg, 2021), in recent years, there has been a notable shift in mental health treatments toward the utilization of digital and online platforms for delivering accessible, cost-effective, and convenient interventions (Wootton, 2016; Nguyen et al., 2022). The COVID-19 pandemic has highlighted the need for remote treatment methods due to lockdowns, social distancing measures, and the growing demand for mental health support (Witteveen et al., 2022). Online and internet-based interventions have gained popularity due to their potential to reach a broader population, overcome geographical barriers, and increase dissemination of evidence-based therapies (Thompson et al., 2021).

However, online group therapy has not yet received sufficient attention in the research literature, and studies focusing on it continue to be limited (Weinberg, 2021). Online and group treatments can combine the advantages of group therapy for increasing access to therapy for more than one person at same time, reducing waiting list time (Pozza and Dèttore, 2017) with the convenience and accessibility of digital platforms that can increase attendance (Khatri et al., 2014; Lopez et al., 2020; Milosevic et al., 2022). Additionally, group treatment conditions can create a unique therapeutic environment that fosters mutual support, the sharing of experiences, and a sense of belonging, which can be particularly beneficial for individuals with AOTDs.

The remote delivery of interventions through web-based and computerized platforms, particularly those based on CBT, are frequently referred as internet-based cognitive behavior therapy (ICBT) or computerized cognitive behavior therapy (CCBT) (Carlbring et al., 2018; Firth et al., 2018). These platforms typically offer interactive modules, virtual therapeutic tools, and self-monitoring features. ICBT has demonstrated potential in treating AOTDs (Firth et al., 2018) by providing structured interventions that participants can complete at their own pace (Andrews et al., 2018; Carlbring et al., 2018). Therapies conducted via video teleconference utilize real-time video communication tools to facilitate interactions between therapists and participants, thus simulating the face-to-face therapeutic experience (Milosevic et al., 2022).

Despite the promise of these forms of therapy in individuals with AOTDs, a comprehensive understanding of the efficacy and feasibility of online group treatments for mental health conditions is needed. The primary objective of this systematic review is to comprehensively explore the efficacy and feasibility of online group treatments for ADs, OCRDs, and TRDs. By synthesizing existing literature, this review aims to provide valuable insights into the effectiveness of online group treatments and address the growing demand for these alternative treatment options in the context of these prevalent mental health conditions. Additionally, this review seeks to identify any potential gaps in the current literature and provide recommendations for future research in this field.

## 2 Materials and methods

### 2.1 Objective

The aim of this review was to systematically evaluate the existing literature on online group therapies for AOTDs to determine the extent of evidence supporting its feasibility and efficacy.

### 2.2 Protocol and registration

This review protocol was registered at the international prospective register of systematic reviews (PROSPERO) under the number CRD42023408491. The reporting of this systematic review was guided by the standards of the preferred reporting items for systematic review and meta-analysis (PRISMA) Statement (Page et al., 2021).

### 2.3 Eligibility criteria

The criteria for study eligibility were established based on key components of the research question using the Participants, Intervention, Comparator, Outcomes, and Study Design (PICOS) framework, as described below.

#### 2.3.1 Participants

The target population comprised adults aged 18 years and above, who received a formal diagnosis of ADs, TRDs, or OCRDs, as per criteria outlined in the DSM or International Classification of Diseases (ICD). The diagnosis had to be assessed using a validated diagnostic interview tool such as mini international neuropsychiatric interview (MINI), structured clinical interview (SCID), composite international diagnostic interview (CIDI) or Anxiety and Related Disorders Interview Schedule (ADIS).

#### 2.3.2 Interventions

This review encompassed all available online group treatments for AOTDs documented in the literature. The criteria for inclusion were group treatments that involved the presence or mediation of a therapist within the group and required active participation of group members. The interventions were required to emphasize interaction among the participants as a fundamental aspect of the protocol, rather than merely suggesting or encouraging their engagement.

#### 2.3.3 Comparator

No control or comparator group was required in this review.

#### 2.3.4 Outcome measures

The primary outcome of interest was to evaluate changes in symptoms related to AOTDs, as measured by formal or validated instruments, both before and after the intervention.

#### 2.3.5 Studies

No filters were applied during the search. However, this review included only randomized and non-randomized controlled trials, observational studies, and case series published in English, Portuguese, German, or Spanish.

### 2.4 Information sources and selection of studies

The electronic systematic search was conducted on 11 March 2023, in the following databases: PUBMED, PsycInfo, Web of Science, and the ClinicalTrials.gov registry. The same search strategy was applied for PUBMED (All Fields), PsycInfo (Any Field), Web of Science (All Fields) and included the following key words: (digital* OR virtual* OR online OR on-line OR tele* OR videoconference OR internet OR remote* OR “user-computer interface” OR mobile OR eHealth) AND (“group intervention” OR “group therapy” OR “group psychotherapy” OR “group cognitive-behavioral therapy” OR “group CBT”) AND (anxiety OR phobi* OR agoraphobia OR panic OR “selective mutism” OR “obsessive-compulsive” OR “body dysmorphic” OR hoarding OR trichotillomania OR “hair pulling” OR “skin picking” OR excoriation OR hypochondri* OR “olfactory reference” OR tic OR tourette OR trauma*).

For the clinical trials, an advanced search was performed using the following terms: (anxiety disorder OR obsessive-compulsive disorder OR obsessive-compulsive related disorder) in “Condition or disease.” (digital* OR virtual* OR online OR on-line OR tele* OR videoconference OR internet OR remote* OR “user-computer interface” OR mobile OR eHealth) in “Other terms” and (“group cognitive-behavioral therapy” OR “group CBT”) in “Intervention/treatment” field, with “All Studies” marked on “Study Status” field.

The screening of the studies was conducted with the Rayyan platform (Ouzzani et al., 2016). Duplicate titles across databases were removed. Three pairs of reviewers (LL-BT, LF-CL and VH-SS) independently reviewed the articles. The screening was performed in two phases (title-abstract and full text) and articles that did not meet the inclusion criteria were excluded. Any disagreement was resolved by discussion during weekly meetings with the six researchers. In the absence of a consensus, a seventh reviewer (LFF) made a final decision. In addition, five independent reviewers performed hand searches of the selected studies’ reference lists to supplement the database searching. Efforts were undertaken to clarify potential confounding aspects by contacting the authors through email.

### 2.5 Assessment of risk of bias in included studies

The assessment of potential bias was conducted independently by two authors (CL and SS) using the (Downs and Black, 1998) for both randomized and non-randomized trials. This checklist examines five domains concerning trial quality: reporting, external validity, internal validity (including bias and confounding), and power. It encompasses 27 items, each scored as “yes” (2 points), “no” (0 points), “partially” (1 point; item 5), or “unable to determine” (0 points; items 11–26). Following Joyce et al. (2018), we incorporated a modified question 27 with responses “yes” (1 point), “no” (0 points), or “not applicable” (0 points). A higher total score reflects greater study quality and reduced risk of bias. The maximum achievable score for the modified checklist was 28. Any discrepancies were resolved through consensus during meetings. Based on the total score, studies were categorized as excellent (> 26), good (20–25), fair (15–19), or poor (< 14) (Joyce et al., 2018).

### 2.6 Data collection process

The research team comprised three psychiatrists (LFF, GM, and VH), two biomedical scientists (MM and SS), and four psychologists (LL, CL, LF, BT). The data extraction was carried out by three dyads of reviewers (LL-BT, LF-CL, and VH-SS), with one reviewer of the dyad responsible for extracting the data and the other reviewer of the dyad conducting the revision of the extracted information. Any disagreement was resolved by discussions within the broad team during weekly meetings. Qualitative and quantitative data were collected using an extraction template. Information regarding the country of study, demographics (age and gender), type of the online group interventions, study design, and primary outcome data (changes in symptoms related to anxiety disorders or obsessive-compulsive disorders) was collected.

### 2.7 Data analysis

The data analysis methodology encompassed a comprehensive narrative synthesis of the included studies. This approach involved presenting the study characteristics, primary and secondary outcomes, using tables and a narrative summary to provide detailed insights into the results.

## 3 Results

**FIGURE 1:**
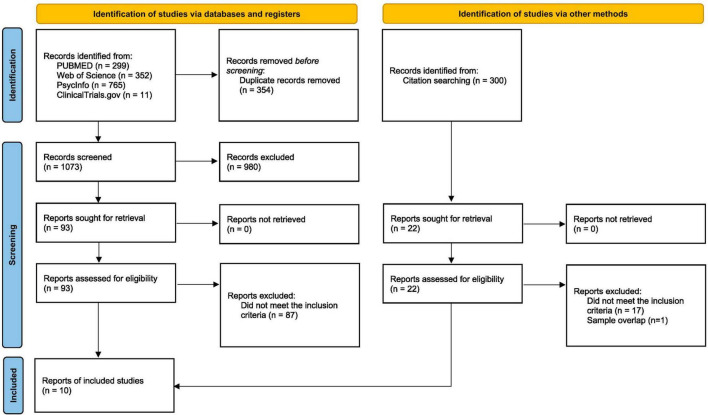
PRISMA diagram (Page et al., 2021).

The search across all databases resulted in a total of 1,427 articles. After removing duplicate references (*n* = 354), 1,073 references underwent screening based on their titles and abstracts. Following this initial screening, 93 reports underwent full-text review. Subsequently, 87 were excluded for not meeting the inclusion criteria, leaving six studies that were finally included. A hand search was conducted on the citations and references of the six included articles to expand the search, resulting in the identification of four additional articles eligible for inclusion. At the end of all screening procedures, ten articles met the eligibility criteria outlined in this review. Research steps are shown in the PRISMA diagram (Figure 1).

Table 1 shows the ten selected articles which were published between 2007 and 2021. Among them, there were five studies of ADs [all of them focusing on subjects with social anxiety disorder (SAD)], four on TRDs [specifically on subjects with post-traumatic stress disorder (PTSD)] and one study with subjects with tic disorders (TD), that was included in this review due to the inclusion of tic disorders within the chapter of OCRDs in ICD-11 (Stein et al., 2020). The studies were conducted at USA (*n* = 6), Australia (*n* = 3) and Central Europe (*n* = 1). The studies were open-label (*n* = 2) and RCTs (*n* = 8), with five of the RCTs being non-inferiority trials.

**TABLE 1:** Sample demographics and clinical variables of selected studies.

References	Country	Design	Diagnostic	Online group therapy	Control	Diagnostic tool	Age, mean (SD)	Female (%)	Major comorbidities
Titov et al., 2008a	Australia	RCT	SAD	IBT (clinician assisted forum)	Waitlist	CIDI-3.0	38.13 (12.24)	58	Not reported
Titov et al., 2008b	Australia	RCT	SAD	IBT (clinician assisted forum)	IBT (self-guided) and waitlist	MINI (DSM-IV)	37.97 (11.29)	61	Not reported
Titov et al., 2009	Australia	RCT-NI	SAD	IBT (clinician assisted forum)	IBT (non-clinician assisted Tel)	MINI (DSM-IV)	38.88 (12.08)	56	Not reported
Schulz et al., 2016	Switzerland Austria and Germany	RCT	SAD	IBT (group clinician assistance)	IBT (individual clinician assistance) and waitlist	SPS; SIAS; SCID-I (DSM-IV)	35.38 (11.16)	53	Specific phobia, MDE, dysthymia, GAD, PDA, OCD, alcohol abuse, substance abuse
Reese et al., 2021	USA	Open-label	TD	IBT (self-guided online MBSR) + VTC	N.A.	SCID (DSM-5)	39.60 (13.10)	20	OCD, ADHD, GAD, SAD, AG, specific phobia, hypochondriasis
Frueh et al., 2007	USA	RCT-NI	PTSD	VTC (CBGT for veterans)	In person (CBGT for veterans)	MINI (DSM-IV); CAPS	VTC: 55 (5) IP: 56 (5)	0	Depressive disorders, anxiety disorders, substance abuse/dependence, psychotic disorder
Morland et al., 2010	USA	RCT-NI	PTSD	VTC (AMT)	In person (AMT)	SCID (DSM-IV); CAPS	54.70 (9.60)	0	Mood, anxiety, substance abuse
Morland et al., 2011	USA	RCT-NI	PTSD	VTC (CPT-C)	In person (CPT)	SCID (DSM-IV); CAPS	48.60 (14.20)	0	Not reported
Morland et al., 2014	USA	RCT-NI	PTSD	VTC (CPT-C)	In person (CPT)	SCID (DSM-IV); CAPS	55.30 (12.50)	0	MDD, anxiety disorder, substance use disorder
Nauphal et al., 2021	USA	Open-label	SAD	VTC (SSRT)	N.A.	ADIS (DSM-5)	36.20 (9.50)	40	GAD, MDD, PDD, AG, specific phobia

ADHD, attention deficit hyperactivity disorder; ADIS, Anxiety and Related Disorders Interview Schedule; AG, agoraphobia; AMT, anger management therapy; CAPS, clinician-administered PTSD scale; CBGT, cognitive-behavioral group therapy; CIDI, composite international diagnostic interview version 3.0; CPT, cognitive processing therapy only with cognitive therapy; DSM-5, Diagnostic and Statistical Manual of Mental Disorders 5 edition; DSM-IV, Diagnostic and Statistical Manual of Mental Disorders 4 edition; GAD, generalized anxiety disorder; IBT, internet-based therapy; MBSR, mindfulness-based stress reduction; MDD, major depressive disorder; MDE, major depressive episode; MINI, mini international neuropsychiatric interview; N.A., not applicable; OCD, obsessive-compulsive disorder; PDA, panic disorder with/without agoraphobia; PDD, persistent depressive disorder; PTSD, post-traumatic stress disorder; RCT, randomized controlled trial; RCT-NI, randomized controlled trial of non-inferiority; SAD, social anxiety disorder; SCID, structured clinical interview for DSM disorders; SD, standard deviation; SIAS, social interaction anxiety scale; SPS, social phobia scale; SSRT, Social Self-Reappraisal Therapy; TD, tic disorder; USA, United States of America; VTC, video teleconference.

### 3.1 Treatments characteristics

In the literature related to online group therapies, there is no consensus regarding the terminologies used to refer to these interventions. Examples of this heterogeneity include terms like videoconferences; telepsychiatry; telehealth; telemedicine; etherapy; internet-, web-, or website-based interventions; and computerized therapy (Barak et al., 2008; Backhaus et al., 2012). To enhance the clarity surrounding online therapy modalities, we have categorized the identified studies into three main groups of interventions: internet-based therapies (IBT), video teleconferencing (VTC), and a combination of both. We provide a definition of these interventions below.

The term “internet-based therapies” (IBT) was employed to characterize self-managed interventions via web-based platforms, with or without asynchronously therapist guidance. These platforms commonly provide interactive modules, virtual therapeutic tools, and self-monitoring features, as well as diverse modes of therapist interaction to guide the therapeutic process, such as e-mail exchanges and forum discussions, where it is applicable.

In contrast, we described as “video teleconferences” (VTC) the online group interventions that incorporate real-time video communication tools for synchronous interaction between therapists or clinicians and participants. We also conventionally referred to all compared interventions conducted face-to-face or in the same room as “in person” and the ones who were not guided by a professional as self-guided treatment.

Most of the selected studies (*n* = 9) provided cognitive-behavioral therapy (CBT) through IBT (*n* = 4) or VTC (*n* = 5), while one of the studies delivered mindfulness-based stress reduction through a combination of IBT with self-guided online lessons and group VTC discussions (Reese et al., 2021). In Tables 2, 3, divided into IBT and VTC, respectively, we have listed the outcomes of the primary diagnosis, as well anxiety, stress, and anger scores at baseline, post-treatment and the last follow up investigated in each study. Additional outcomes are detailed in the text.

**TABLE 2:** IBT primary clinical outcomes.

Study	References	Design	Quality*	Severity scales	Online group therapy			Control			Conclusion
					Pre-treatment	Post-treatment	Follow up	Pre-treatment	Post-treatment	Follow up	
#1	Titov et al., 2008a	RCT	17/28		IBT			Waitlist			IBT clinician assisted group has much greater significantly reduced symptoms of social phobia than waitlist.
				SPS	34.02 (14.42)	20.64 (10.46)	N.A.	36.08 (16.63)	33.92 (14.70)	N.A.	
				SIAS	53.82 (11.29)	39.24 (12.18)		54.67 (12.41)	50.59 (14.15)		
#2	Titov et al., 2008b	RCT	16/28		IBT			IBT (self-guided) and waitlist			The clinician assisted condition was superior to the self-guided and waitlist, with no differences between self-guided and waitlist condition, regarding reducing social phobia symptoms.
				SPS	34.71 (15.04)	18.65 (N.R.)	N.A.	SG: 32.87 (17.02) WL: 34.38 (18.77)	SG: 28.27 (16.27) WL: 35.44 (N.R.)	N.A.	
				SIAS	54.71 (10.59)	40.87 (N.R.)		SG: 52.50 (9.30) WL: 52.09 (13.60)	SG: 48.03 (13.59) WL: 53.06 (N.R.)		
#3	Titov et al., 2009	RCT-NI	17/28		IBT (Forum)			IBT (Tel)			The clinician assisted condition and technician assisted condition had a large effect size on post-treatment regarding reducing social phobia symptoms but was not significant different between each other
				SPS	35.74 (10.15)	18.82 (12.14)	N.A.	35.70 (13.24)	20.88 (12.61)	N.A.	
				SIAS	54.59 (10.17)	37.56 (11.56)		54.26 (12.21)	35.26 (13.57)		
#4	Schulz et al., 2016	RCT	20/28		IBT (group)			IBT (individual) and waitlist			At post-treatment, both active conditions showed superior outcome regarding reducing SAD symptoms than waitlist, with no significant difference between the two active conditions in symptom reduction.
				SPS	38.90 (14.04)	23.78 (13.16)	20.66 (10.49)	IT: 39.32 (11.64) WL: 37.35 (12.45)	IT: 21.07 (10.94) WL: 34.58 (12.30)	IT: 20.61 (11.85) WL: N.R	
				SIAS	50.93 (14)	36.56 (16.01)	34.28 (16.09)	IT: 50.48 (14.48) WL: 50.97 (13.58)	IT: 33.87 (14.47) WL: 47.67 (10.97)	IT: 32.36 (15.38) WL: N.R.	

IBT, internet-based therapy; N.A., not applicable; RCT, randomized controlled trial; RCT-NI, non-inferiority RCT; SG, self-guided treatment; SIAS, social interaction anxiety scale; SPS, social phobia scale; Tel., Telephone; WL, waitlist;

*Risk of bias analysis = paper score / total score = excellent (> 26), good (20–25), fair (15–19), or poor (< 14).

**TABLE 3:** VTC primary clinical outcomes.

Study	References	Design	Quality*	Severity scales	Online group therapy			Control			Conclusion
					Pre-treatment	Post-treatment	Follow up	Pre-treatment	Post-treatment	Follow up	
#5	Frueh et al., 2007	RCT-NI	19/28		VTC			In person			VTC was “as good as” in person, with low rates of clinical change in both groups.
				PCL-M	67.0 (9.4)	68.1 (11.0)	61.4 (14.6)	62.3 (12.8)	56.5 (10.1)	60.5 (9.8)	
#6	Morland et al., 2010	RCT-NI	19/28		VTC			In person			Participants in both conditions showed substantial improvement at posttreatment regarding to anger and PTSD symptoms with a non-inferiority result for the VTC to in person regarding anger symptoms.
				PCL-M	64.50 (11.60)	59.20 (15)	N.A.	65.80 (10.80)	57.40 (16)	N.A.	
				STAXI-2 (AE)	56.70 (12)	42.40 (16.20)	42 (15.60)	55 (10.3)	46.60 (12.20)	46.60 (15.30)	
				STAXI-2 (TA)	28 (6)	22.10 (6.20)	22.40 (7.30)	27.80 (5.60)	23.30 (6)	25.60 (8.20)	
				NAS-T	109.30 (16.10)	94.20 (19.10)	97.70 (20.20)	109.8 (14)	99.20 (17.10)	101 (22.50)	
#7	Morland et al., 2011	RCT-NI	16/28		VTC			In person			Both groups showed clinically meaningful reductions in PTSD symptoms and no significant between-group differences on clinical or process outcome variables.
				CAPS	82.50 (N.R.)*	69 (N.R.)*	59 (N.R.)*	77 (N.R.)*	62 (N.R.)*	66 (N.R.)*	
#8	Morland et al., 2014	RCT-NI	17/28		VTC			In person			Clinical outcomes found VTC to be noninferior to in person treatment with significant reductions in PTSD symptoms at posttreatment and maintained at 3 and 6 mo. follow-up.
				CAPS	72 (14.60)	55.60 (18.80)	56.20 (18)	68.90 (13)	58.70 (21)	57.80 (18.7)	
#9	Nauphal et al., 2021	Open-label	11/28		VTC			N.A.			All group members reported decreases in social anxiety with two participants demonstrated statistically significant and clinically meaningful decreases in their scores on the SPIN from baseline to posttreatment.
				SPIN	33.40 (16)	15.80 (12.91)	N.A.				
				OASIS	6.40 (3.21)	3.20 (2.77)					
				DASS-21(A)	5.60 (8.71)	1.20 (1.09)					
				DASS-21(SR)	11.20 (10.90)	7.20 (7.29)					
#10	Reese et al., 2021	Open-label	10/28		IBT + VTC			N.A.			It was reported a modest or subdued reduction in the tic symptoms
				YGTSS (TTS)	31.2 (5.9)	29.4 (6.5)	N.A.				
				YGTSS (IPM)	32.0 (4.4)	28.0 (4.4)					

CAPS, clinician- administered PTSD scale; DASS-21(A), Depression Anxiety Stress Scales (anxiety subscale); DASS-21(SR), Depression Anxiety Stress Scales (stress reactivity subscale); IBT, internet-based therapy; N.A., not applicable; NAS-T, Novaco anger scale-total score; OASIS, overall anxiety severity and impairment scale; PCL-M, PTSD checklist-military version; PTSD, post-traumatic stress disorder; RCT, randomized controlled trial; RCT-NI, non-inferiority RCT; SPIN, social phobia inventory; STAXI-2 (AE), State-Trait Anger Expression Inventory-2 (anger expression); STAXI-2 (TA), State-Trait Anger Expression Inventory-2 (trait anger); VTC, video teleconference; YGTSS (IPM), Yale Global Tic Severity Scale (impairment); YGTSS (TTS), Yale Global Tic Severity Scale (total tic severity);

*Risk of bias analysis = paper score / total score = excellent (> 26), good (20–25), fair (15–19), or poor (< 14). *Study #7 presented data on median scores.

### 3.2 Internet-based therapies (IBT)

Four RCTs that utilized IBT “enhanced” by online forum discussions were identified, all of which employed CBT based treatments for SAD. The decision to include these studies was based on the researchers’ judgment that this online forum discussions represented some sort of group interventions. The four studies included in this section compared a clinician assisted, “forum-enhanced” internet-based CBT to (i) a waiting list (Titov et al., 2008a) (ii) a waiting list and a self-guided, “forum-enhanced” internet-based CBT (Titov et al., 2008b) (iii) a non-specialist, “telephone enhanced” individual internet-based CBT (Titov et al., 2009) and (iv) a waiting list and clinician assisted, “email enhanced” individual internet-based CBT (Schulz et al., 2016).

The first three studies investigating IBT for SAD were conducted by the same research group (Titov et al., 2008a,b, 2009) with the intervention protocol termed the “Shyness Programme,” a clinician assisted treatment consisting of online lessons. During this treatment participants completed six online CBT based lessons with cognitive-behavioral homework assignments. Participants could also participate in an online discussion forum and contact a therapist through email. These three studies involved independent samples (Titov, personal communication).

#### 3.2.1 Study 1

The first RCT, named *Shyness 1* (Titov et al., 2008a) compared IBT assisted by group forum to a waitlist condition. The aim was to access the effectiveness of this intervention, assisted by a forum discussion and e-mail contact with therapist, in a group of 105 participants that were randomly assigned to the two conditions. They reported significant difference between study groups regarding reductions in the two measures of social anxiety symptoms [social interaction anxiety scale (SIAS: *p* < 0.001) and the social phobia scale (SPS: *p* < 0.001)]. A comparable pattern was noted in the level of disability and psychological distress. Change in social anxiety symptoms was linked to a decrease in disability and psychological distress. Both groups showed reductions after treatment with no significant difference between them in relation to depressive symptoms (*p* > 0.05).

The *Shyness 1* study also reported improvements across all measures in the waitlist control group, which is likely to have reduced the differences between groups. The outcomes derived from the treatment satisfaction questionnaire demonstrated a considerable level of contentment with the treatment, as all participants indicated they were either “very satisfied” or “mostly satisfied.” More than 97% of participants rated the quality of the treatment modules as “excellent” or “good,” and 93% rated the quality of the online correspondence with the therapist as “excellent” or “good.” The average number of completed lessons was 5.2 (out of a total of 6), with 39 participants successfully completing all six lessons. The overall completion rate reached 78%. In terms of dropouts, 12 participants did not finish the post-treatment questionnaires or withdrew before starting the program.

#### 3.2.2 Study 2

The *Shyness 3*, another RCT of IBT conducted by Titov et al. (2008b), examined whether participants could successfully complete the program independently. The study compared the same clinician assisted IBT from *Shyness 1* versus a self-guided treatment version, where therapists did not communicate by email and refrained from intervening in the forum group (they solely monitored the participants’ progress) and waitlist. This three-arm Australian study randomly allocated a total of 98 individuals with SAD into the two active treatment conditions (clinician-assisted *n* = 32 or self-guided *n* = 31) and a waitlist (*n* = 35) group. They show that the clinician-assisted group presented significantly greater improvement in SAD symptoms (SPS and SIAS instruments) compared to both waitlist and self-guided groups (*p* < 0.001). However, no significant difference was observed between waitlist and self-guided conditions. The study results suggest the effectiveness and reliability of this IBT program for SAD.

The secondary outcomes analysis presented in this study indicated a significant difference for group on disability according to the Sheehan disability scale (SDS: *p* < 0.001), but not for depression symptoms (*p* > 0.05) or psychological distress (*p* > 0.05). Post-hoc pairwise comparisons of the groups presented a notable distinction between the clinician assisted and waitlist concerning the SDS (*p* < 0.003), while no significant differences were observed between the clinician-assisted and self-guided groups, or between the self-guided and waitlist conditions in terms of SDS. The same pattern was shown about the estimate of avoidance, with a marginally significant difference between clinician-assisted and waitlist groups in avoidance (*p* < 0.003), but not between clinician-assisted and self-guided, or self-guided and waitlist conditions (*p* > 0.05). Regarding estimate of attendance, no significant group difference was found at this study (*p* > 0.05).

Additionally, 97% of participants in the clinician assisted “forum enhanced” group reported a significantly higher level of satisfaction with the overall treatment (either “very satisfied” or “mostly satisfied”) compared to 62% of the self-guided “forum enhanced” condition (*p* < 0.01). A total of six individuals did not finalize the post-treatment questionnaires or withdrew before the program’s initiation (one at clinician-assisted, four at self-guided and one at waitlist condition). Twenty-four participants in the clinician-assisted group (77%) and 10 participants in the self-guided group (33%) successfully finished all six lessons within the stipulated time frame. The mean number of completed lessons was notably greater in the clinician-assisted condition, with a value of 5.39 (SD = 1.31), compared to 3.97 (SD = 1.87) in the self-guided group (*p* < 0.001).

#### 3.2.3 Study 3

The final Titov’s study included in this review is called *Shyness 6* (Titov et al., 2009). It was a non-inferiority RCT that aimed to compare the therapeutic benefits and acceptability of two distinct modes of guidance, clinician-assisted discussion forum (IBT + Forum) compared to telephone call from a technician (IBT + Tel), testing the benefits of a weekly telephone call by a non-specialized assistant. In the telephone treatment condition, participants were called weekly by a technician who provided commendations and encouragement to persevere in a short call, but no clinical advice was offered. On the other hand, participants in the forum group were invited to post-messages about their progress and questions on a series of online discussion forums moderated by a clinician. The clinician read and responded to forum messages each Monday, Wednesday, and Friday. Both groups received identical internet-based treatments delivered through computer.

A total of 85 participants were randomized for this later study, with 42 individuals assigned to the condition receiving IBT with a clinician forum discussion support and 43 individuals assigned to the condition receiving IBT with non-clinician telephone support over an eight weeks program. The study did not find any significant difference between the two groups concerning social anxiety symptoms (SIAS: *p* < 0.38 and SPS: *p* < 0.37) at the post-treatment assessment. They also found that both treatments resulted in good clinical outcomes with equivalent patient acceptability and indicated that more than one type of contact with patients may be effective and acceptable. Similar results were also found regarding the completion of the treatment modules, with both groups exhibiting a completion rate of 79% of participants who successfully concluded all six modules within the designated timeframe. Participants from both groups completed around five lessons. Secondary outcomes data from depression, disability and psychological distress failed to show any significant posttreatment differences between groups.

Eventually, a total of nine participants dropped out of the study (3 from clinician assisted group and 6 from technician assisted group) did not completing the posttreatment questionnaires or withdrawing before beginning the program. Among them, the three withdrew from the clinician condition were before treatment. The results of this RCT demonstrated that both versions of the Shyness program, enriched with additional resources, frequent reminders, either weekly telephone called or accessing to a clinician-assisted forum, can lead to increased completion rates, improved clinical outcomes, and high acceptability ratings.

#### 3.2.4 Study 4

Finally, Schulz et al. (2016) conducted a three-arm RCT (*n* = 149) of a CBT internet-based wherein participants were randomly assigned to either of two active conditions: clinician assisted “forum enhanced” group IBT or clinician assisted “email enhanced” individual IBT, or alternatively placed on a waiting list. The objective was to assess the effectiveness of an IBT group treatment (GT) which involved clinician-assisted forum discussions, in reducing symptoms of social anxiety. This was compared to a waitlist (WL) control group and an IBT clinician assisted individual treatment (IT) through email contact. All participants utilized identical self-managed materials for SAD, which consisted of eight text-based sessions to be completed sequentially. Participants were advised to engage in one session per week and to practice the exercises repeatedly throughout the 12-week treatment period.

Their finds suggests that SAD can be successfully treated with a clinician assisted IBT, conducted by group (forum discussions) as much as an individual (e-mail contact) condition (SPS: *p* = 0.63 and SIAS: *p* = 0.99). Furthermore, there were significant time effects observed at pre-, post- and the six-month follow-up treatments time points on both groups, indicating that the treatments conditions benefits were maintained by most participants over an extended duration of time regarding social anxiety, depression, global severity, interpersonal problems and mental health improvements (all of them with *p* < 0.001).

The two active conditions also did not differ regarding the dropout rates (25% before the posttreatment assessment). Additionally, the high ratings of client satisfaction indicate that the group-guided format was well received, as much as the individual condition. Although no significant differences were identified between the group treatment and individual treatment formats in terms of improvements in social anxiety symptoms, diagnostic response, or attrition rates, a notable distinction emerged in the amount of time invested by supporting clinicians in providing assistance. On average, patients receiving individual assistance required three times more the clinician’s time compared to those using the group format.

### 3.3 Video teleconference treatments (VTC)

A total of five studies have been conducted with VTC being the main intervention evaluated (Frueh et al., 2007; Morland et al., 2010, 2011, 2014; Nauphal et al., 2021). All studies by Morland et al. involved independent samples (personal communication). Some studies specified that the participants were physically present in the same room, while the therapist or clinician was the only one attending through VTC (Morland et al., 2010, 2011, 2014). In contrast, other study conducted during the COVID-19 pandemic involved all participants and the therapist being distant from each other through zoom VTC (Nauphal et al., 2021). Frueh et al. (2007) did not specify this aspect of VTC, i.e., they only reported that it was done through “PC-based videoconferencing equipment (Via-Video, Polycom).”

#### 3.3.1 Study 5

In 2007, a noninferiority RCT was undertaken in the US to investigate the effectiveness of a VTC CBT-based intervention for combat-related PTSD (Frueh et al., 2007). The study aimed to compare two distinct modes of group therapeutic service delivery: telepsychiatry via VTC and in-person sessions conducted in the same room. A total of 38 participants were randomized (17 into VTC, 21 into in-person). All patients participated in a group CBT program specifically designed for veterans with PTSD, known as Social and Emotional Rehabilitation. The program comprised 14 weekly, 90-min treatment sessions focused on targeted social skills training and engaging in activities aimed at enhancing social participation.

There were low rates of clinical improvement in both groups and no significant differences were observed between the groups in terms of any clinical outcome (PTSD: *p* = 0.39, depressive symptoms: *p* = 0.65, global severity: *p* = 0.97, quality of social relationships: *p* = 0.70, social activities outside: *p* = 0.83 and inside: *p* = 0.43). The only reportable notable difference at post-treatment was that the in-person group reported feeling more comfortable while communicating with their therapist (*p* = 0.03). In the VTC group, eight participants (47%) did not complete the active treatment phase, whereas nine participants (43%) in the in-person group also did not complete the treatment phase (*p* ≥ 0.05). Session attendance did not show any significant differences between the groups. However, the in-person group had a higher likelihood of completing their homework assignments (*p* = 0.04). In sum, Frueh et al. (2007) findings suggests that a VTC group treatment is as efficacious as traditional care in person group treatment.

### 3.3.2 Study 6

Morland et al. (2010) conducted a noninferiority randomized controlled trial involving 125 male rural combat veterans with PTSD. The purpose was to establish the noninferiority of a telemedicine method by VTC (*n* = 61) in comparison to the conventional in-person (*n* = 64) cognitive-behavioral anger management group therapy (AMT). In both treatment conditions, the therapist was the only one who was either remote or physically present, while the participants remained in the same room within the clinic. Both groups underwent the same manual-based CBT 12-session AMT protocol, with 2 sessions per week over a 6-week period.

Authors found that both groups of participants experienced significant decreases in anger symptoms with posttreatment, 3 and 6 months follow up (effect sizes ranging from 0.12 to 0.63). Participants in the VTC condition demonstrated a reduction in anger symptoms similar (“non-inferior”) to symptom reductions in the in-person groups. The authors demonstrated a significant reduction in PTSD symptoms (PCL-M) after treatment in both the VTC and in-person conditions. However, there were no difference between the two conditions based on the PCL-M outcomes.

There was no significant difference between the VTC and in-person conditions in terms of the process outcomes (i.e., satisfaction; expectancy; attendance or homework completion). However, participants in the in-person condition reported a higher overall group therapeutic alliance compared to VTC participants (*p* = 0.02). The dropout rates also did not differ between the two treatment groups (*p* = 0.43). This data suggested the feasibility of implementing this online group intervention for this specific population as good as the in-person traditional treatment.

#### 3.3.3 Study 7

Another VTC study found in this review (Morland et al., 2011) presented a preliminary pilot data, in the form of a brief report paper, from a larger RCT ongoing at the time of publication. The study provided initial clinical and feasibility data that assesses the effectiveness of group CBT for PTSD delivered through VTC in comparison to in-person delivery. Similarly to the previous study, a group of male veterans with combat-related PTSD residing in rural areas of Hawaii (USA) were assigned to treatment groups that occurred at the same room in the same clinic. In both treatment conditions, the therapist was the only one who was either remote or physically present, while the participants remained in the same room.

In this pilot study, 13 PTSD veterans were enrolled and randomly assigned for the two conditions (VTC: *n* = 6; in-person: *n* = 7) and 11 participants completed therapy. The two treatment groups followed the same cognitive processing therapy with cognitive therapy only (CPT-C), a protocol of twelve 90-min sessions that took place twice a week over a 6-week period and were delivered in parallel. The analysis of the PTSD clinical outcome data [Clinician-Administered PTSD Scale (CAPS) instrument] supported the effectiveness of this treatment with this specific population independently of the modalities (VTC or in person). Wilcoxon signed rank tests demonstrated significant variations between CAPS scores before treatment compared to post-treatment (*p* = 0.004), and at the 6-month follow-up (*p* = 0.005), with no significant differences between the treatment conditions neither at post-treatment (*p* > 0.05) nor at the 6-month follow-up (*p* > 0.05). These results provide initial endorsement for the clinical efficacy of this group VTC for PTSD.

During the active treatment phase, only one participant (15%) dropped out (from the VTC group). Both conditions reported elevated levels of treatment credibility, satisfaction with their care, and adherence to assigned homework tasks. However, there was no significant difference between the in-person and VTC conditions in terms of treatment dropout (*p* = 0.26), in the number of sessions attended (*p* > 0.05) and in total number of completed homework assignments (*p* > 0.05). This data implies that this VTC group intervention is just as feasible and acceptable as the in-person conventional intervention.

#### 3.3.4 Study 8

Another study conducted by the same group of authors (Morland et al., 2014), a noninferiority RCT, compared the clinical and process outcomes of CPT-C delivered via VTC and in-person conditions. A total of 125 participants were randomly divided into two conditions of group interventions (in person: *n* = 64 and VTC: *n* = 61) within a rural sample of veterans with PTSD. Similar to the previously mentioned study, participants underwent the manualized CBT based group CPT-C protocol that involved twelve 90-min sessions held twice a week over a duration of 6 weeks. A noninferiority design to evaluate the impact of delivery modality on PTSD symptoms tested the hypothesis that VTC is noninferior to in-person condition.

The results suggest that both groups (VTC and in-person) experienced significant reductions in PTSD symptoms scores over time (CAPS: *p* < 0.05 for each), but there was no statistically significant difference between the groups at the analyzed follow-up points. Despite improvements observed in both groups, a clear difference between the two treatment approaches regarding PTSD could not be established. There was no significant difference between the in-person (*n* = 7) and VTC (*n* = 12) conditions in terms of the number of veterans who dropped out (15.2%) between randomization and the first session (*p* = 0.24).

Furthermore, there were no significant differences between the groups in terms of the number of attended sessions (*p* = 0.58), the percentage of patients completing the treatment (*p* = 0.72), and homework completion (*p* = 0.79). Compliance with the treatment was also strong, with an average attendance of 9.9 out of 12 sessions, and 76.8% completing the essential treatment course of a minimum of 10 sessions. In both conditions, participants indicated elevated levels of treatment credibility, contentment with the care received, and adherence to homework tasks, along with a strong sense of alliance with the therapist and fellow group members.

#### 3.3.5 Study 9

The most recent studies involving a VTC intervention in this review were open-label (Nauphal et al., 2021; Reese et al., 2021). The Nauphal et al. (2021) study investigated the acceptability, feasibility, and initial effectiveness of delivering group CBT for SAD through VTC. The group met remotely through zoom for eight sessions with 2 h each, the treatment consisted of an adapted Social Self-Reappraisal Therapy (SSRT) protocol of CBT based intervention. All group participants (*n* = 5) engaged in treatment-related tasks and completed homework assignments. Four members attended all eight sessions, while one member attended seven sessions, but missed the final one.

According to the authors, all group members reported decreases in social anxiety (*d* = 1.07), with two participants exhibiting statistically and clinically significant reductions in their SAD symptoms scores from baseline to post-treatment. At the group level, changes in anxiety and related impairment displayed a moderate to large magnitude (*d* = 0.78). Changes in anxiety itself presenting a moderate magnitude (*d* = 0.56), and stress-related symptoms showed a decrease in symptom severity during treatment (*d* = 0.65), *p*-values not reported. In a broader context, there was a decrease in depression symptoms from the pre-treatment to the post-treatment phase. One of the participants who had not reported noteworthy depression before treatment experienced an upsurge in depressive symptoms after the intervention.

The data reported in the study regarding overall satisfaction and satisfaction with VTC treatment indicate high levels of contentment with the intervention, even with the quality of remote service delivery. The participants found the VTC treatment format to be acceptable and sufficiently met their requirements. Their conclusion was that the results provided promising support for the acceptability, feasibility, and effectiveness of VTC group CBT for SAD, although additional research would be needed to establish efficacy and cost-effectiveness.

### 3.4 Combined IBT and VTC treatments

#### 3.4.1 Study 10

Reese et al. (2021) aimed to investigate the feasibility, acceptability, and safety of an online mindfulness based (MBSR) group intervention designed for adults with tic disorders (*n* = 6). Five of them completed treatment, all participants completers meeting DSM-5 diagnostic criteria for a primary diagnosis of tourette syndrome at baseline. This final study integrates 1.5-h of self-guided online lessons conducted over 8 weeks, supplemented by a 1-h therapist-guided group session via VTC each week. This intervention also involves home practice and appears to amalgamate both previously reported modalities, as it combines a self-administered component of online lessons (an IBT component) with therapist guided VTC.

Authors observed a mean reduction of 1.8 points (SD = 2.04) in tic severity and a mean reduction of 4 points (SD = 5.48) in tic-related impairment with the Yale Global Tic Severity Scale (YGTSS) (statistical significance *p*-value not reported). Reese et al. (2021) reported high level of satisfaction with the intervention measured by the Client Satisfaction Questionnaire (CSQ). Participants attended 87.5% of all scheduled VTC sessions. One individual withdrew after the screening assessment due to personal reasons. Once treatment began, no participants dropped out. No serious adverse events were reported and none of the reported events were deemed to be connected to the intervention (e.g., strained muscle, back pain, knee injury). These findings suggest that MBSR delivered through a mix of IBT and VTC is acceptable, feasible in its implementation and safe, with some adjustments to be made for enhancing adherence.

### 3.5 Risk of bias–quality assessment

Most of the studies were categorized as fair quality (Frueh et al., 2007; Titov et al., 2008a,b, 2009; Morland et al., 2010, 2011, 2014). Two studies received a poor quality rating (Nauphal et al., 2021; Reese et al., 2021), while one study was rated as good quality (Schulz et al., 2016). In Tables 2, 3 we listed the quality scores of the ten studies.

## 4 Discussion

Online group therapies may be useful for being more affordable than individual therapies and available for a greater number of individuals, particularly AOTDs patients who are avoidant and have problems leaving home for displaying incapacitating social fears (e.g., SAD), contamination concerns (e.g., OCD) or preoccupations with further traumatic events (e.g., PTSD). This form of treatment may also be predominantly useful for individuals with AOTDs living in remote areas, particularly when facing “lockdown” measures during the aggravation of pandemics or other similar disasters.

In this study we were able to perform a comprehensive, “transdiagnostic” and systematic review on the efficacy of online group therapies on populations with different type of AOTDs. Broadly speaking, we found evidence supporting the efficacy of IBT and VTC in different scenarios, particularly in populations with SAD (Titov et al., 2008a,b, 2009; Schulz et al., 2016; Nauphal et al., 2021), PTSD (Frueh et al., 2007; Morland et al., 2010, 2011, 2014) and Tourette syndrome (Reese et al., 2021). Of note, at least half of these studies involved non-inferiority designs, and tended to demonstrate similar efficacies of online therapies to that of more conventional forms of treatment delivery. We will try to summarize and discuss the findings of these studies below.

Firstly, we found that four RCTs tested the efficacy of IBTs (i.e., computerized CBTs with clinically assisted online forums) in participants with SAD. Since these online forums stimulated an active interaction between participants, they were considered online group therapies. These studies showed computerized CBTs with clinically assisted online forums to be superior to waiting lists (Titov et al., 2008a,b; Schulz et al., 2016) and not inferior to similar versions that were also “forum enhanced” but self-guided (i.e., not clinician-assisted), “telephone enhanced” by a contact with a non-specialist (Titov et al., 2009), and “email-enhanced” by a contact with a clinician (Schulz et al., 2016). Thus, one of the main findings of this review is the demonstration of the efficacy of the specific “forum enhanced” protocol by Titov and its self-guided version in individuals with SAD.

Also, four additional RCTs tested the efficacy of different forms of CBT-based interventions delivered through VTC for patients with combat-related PTSD, often from rural areas. These protocols included packages that involved social and emotional rehabilitation strategies (Frueh et al., 2007), anger management (Morland et al., 2010), and cognitive processing therapy (Morland et al., 2011, 2014). These trials found VTC protocols to be as effective for PTSD symptoms as corresponding in-person sessions. Two remaining VTC trials were open and demonstrated the effectiveness of SSRT in SAD (Nauphal et al., 2021) and of an online MBSR group intervention in Tourette syndrome (Reese et al., 2021).

Methodological differences among VTC studies, which may or may not be relevant for their accurate interpretation, encompass factors like whether participants were physically present in the same room (Morland et al., 2010, 2011, 2014) or were distant from each other and maintain contact through Zoom or similar programs (Nauphal et al., 2021; Reese et al., 2021). For instance, it’s possible that being in the same physical room with other participants while the therapist delivers treatment remotely could closely simulate the dynamics of traditional group therapies, in comparison to therapeutic groups where participants are located in their respective homes and interact through an online platform.

Despite showing the utility of specific therapies for selected AOTDs, our review also identified some relevant gaps in the literature. Most importantly, the coverage of studies investigating online group treatments for AOTDs seems restricted, as no study was found in conditions as common as OCD, panic disorder, and specific phobias, among others. Also, we have not found homogeneity in research terms (e.g., videoconferences; telepsychiatry; telehealth; e-therapy; telemedicine; internet-based interventions; computerized therapy), characteristics of the interventions, and study designs in the area. For instance, for the purposes of the present review, treatments that were aided by online forums were considered forms of group therapies for having some interaction between subjects. However, whether these strategies truly qualify a certain treatment as group may be debatable.

Our study has some additional limitations. For instance, as one of the main strengths of online group treatments is increased scalability to broader populations, the restriction of our review to studies including samples diagnosed according to clinician-based interviews (and excluding participants who reported clinically significant AOTDs symptoms based on self-report measures) may be considered misaligned with our initial objectives. However, it is also important to review the efficacy of online group treatments in well characterized samples. Also, despite its undeniable scalability and transdiagnostic benefits (Alvarez-Jimenez et al., 2014; De Jaegere et al., 2019; Krysta et al., 2021; Shorey et al., 2021), it may be challenging to employ online group treatments for AOTDs in the context of specific clinical conditions like intellectual disability, psychosis, autism, or increased suicidality.

To sum up, our systematic review identified studies which support the efficacy of online group therapies for individuals with SAD and PTSD. More specifically, a handful of RCTs confirmed the usefulness of IBT in individuals with SAD and of VTC in individuals with PTSD. However, it still can be argued, for instance, that these studies tend to come from the same laboratories, and that further studies from different research groups may be needed to replicate the use of these and other forms of online treatments in individuals with SAD, PTSD, and other clinical populations, such as OCD, panic disorder, agoraphobia and specific phobias.

## Data availability statement

The original contributions presented in this study are included in this article/supplementary material, further inquiries can be directed to the corresponding author.

## Author contributions

LL: Conceptualization, Data curation, Formal analysis, Investigation, Writing – original draft, Project administration, Writing – review & editing. SS-R: Conceptualization, Data curation, Formal analysis, Investigation, Writing – original draft, Resources. MM-d-O: Conceptualization, Supervision, Writing – review & editing, Investigation. CL: Conceptualization, Data curation, Formal analysis, Investigation, Writing – original draft. VH: Conceptualization, Data curation, Investigation, Writing – original draft. BT: Data curation, Investigation, Writing – original draft. LF: Data curation, Investigation, Writing – original draft. GM: Conceptualization, Data curation, Investigation, Project administration, Supervision, Writing – review & editing. LFF: Conceptualization, Data curation, Investigation, Supervision, Writing – original draft, Writing – review & editing.
